# Bridging Real-World Data Gaps: Connecting Dots Across 10 Asian Countries

**DOI:** 10.2196/58548

**Published:** 2024-08-15

**Authors:** Guilherme Silva Julian, Wen-Yi Shau, Hsu-Wen Chou, Sajita Setia

**Affiliations:** 1 Pfizer Brazil São Paulo Brazil; 2 Pfizer Corporation Hong Kong Limited Hong Kong China (Hong Kong); 3 Pfizer Ltd Taipei Taiwan; 4 Executive Office, Transform Medical Communications Limited Wanganui New Zealand

**Keywords:** Asia, electronic medical records, EMR, health care databases, health technology assessment, HTA, real-world data, real-world evidence

## Abstract

The economic trend and the health care landscape are rapidly evolving across Asia. Effective real-world data (RWD) for regulatory and clinical decision-making is a crucial milestone associated with this evolution. This necessitates a critical evaluation of RWD generation within distinct nations for the use of various RWD warehouses in the generation of real-world evidence (RWE). In this article, we outline the RWD generation trends for 2 contrasting nation archetypes: “Solo Scholars”—nations with relatively self-sufficient RWD research systems—and “Global Collaborators”—countries largely reliant on international infrastructures for RWD generation. The key trends and patterns in RWD generation, country-specific insights into the predominant databases used in each country to produce RWE, and insights into the broader landscape of RWD database use across these countries are discussed. Conclusively, the data point out the heterogeneous nature of RWD generation practices across 10 different Asian nations and advocate for strategic enhancements in data harmonization. The evidence highlights the imperative for improved database integration and the establishment of standardized protocols and infrastructure for leveraging electronic medical records (EMR) in streamlining RWD acquisition. The clinical data analysis and reporting system of Hong Kong is an excellent example of a successful EMR system that showcases the capacity of integrated robust EMR platforms to consolidate and produce diverse RWE. This, in turn, can potentially reduce the necessity for reliance on numerous condition-specific local and global registries or limited and largely unavailable medical insurance or claims databases in most Asian nations. Linking health technology assessment processes with open data initiatives such as the Observational Medical Outcomes Partnership Common Data Model and the Observational Health Data Sciences and Informatics could enable the leveraging of global data resources to inform local decision-making. Advancing such initiatives is crucial for reinforcing health care frameworks in resource-limited settings and advancing toward cohesive, evidence-driven health care policy and improved patient outcomes in the region.

## Introduction

Real-world data (RWD) in medical and health research describes data relating to patients’ health status or the delivery of health care in an environment outside of conventional clinical trials. This includes data routinely collected for treatment and disease registries, electronic medical records (EMRs), insurance claims, and other health databases that collect information reported by patients or health care professionals [1,2]. Extending from this concept of RWD is real-world evidence (RWE), which is the analyses produced from appropriate, well-designed studies using RWD [3].

In randomized controlled trials, patients with severe forms of disease or multiple comorbidities are typically excluded from participating due to potential risks or adverse events [2]. This leads to a large number of underrepresented patients, reducing the generalizability of interventions in the population, particularly for those who may need it most. RWD can provide the external validity needed when exploring different interventions and treatment strategies across the health care system [2]. To provide external validity for different interventions and disease management strategies, different study styles are used for RWD generation. These can be broadly categorized as comparative effectiveness research (CER) and descriptive studies (non-CER) [4]. CER can be defined as studies that primarily compare interventions and strategies, while non-CER are observational studies that aim to provide a descriptive overview of factors such as disease prevalence or treatment patterns [4,5].

Two scoping reviews have been conducted to explore the spectrum of RWE from linked databases in Asia and their possible impact on health care evolution, strategy, and policy [6,7]. The literature search for these reviews was conducted on PubMed in September 2022 and May 2023, and analysis of RWD publications across these countries assumed a linear distribution of studies [6,7]. In the initial scoping review, the trends and research warehouses for RWD-published studies from 3 countries in Asia with varying health care systems, Taiwan, India, and Thailand, were evaluated. The second scoping review continued this explorative research in 7 other diverse Asian health care systems using the established protocol from the prior review [4,7]. This study aimed to understand the evolving landscape of RWD use and its implications across Hong Kong, Indonesia, Malaysia, Pakistan, the Philippines, Singapore, and Vietnam. The number of total publications and single-country studies (SCS) and cross-country collaboration studies (CCCS) from all the countries in these scoping reviews was used to archetype them as either “Solo Scholars” if they published predominantly SCS with relatively higher number of studies published in last 5 years or “Global Collaborators” if they published less numbers and predominantly CCCS [7]. Hong Kong, India, Malaysia, Singapore, Taiwan, and Thailand were categorized as Solo Scholars, and Indonesia, Pakistan, the Philippines, and Vietnam were categorized as Global Collaborators (Multimedia Appendix 1).

**Figure 1:**
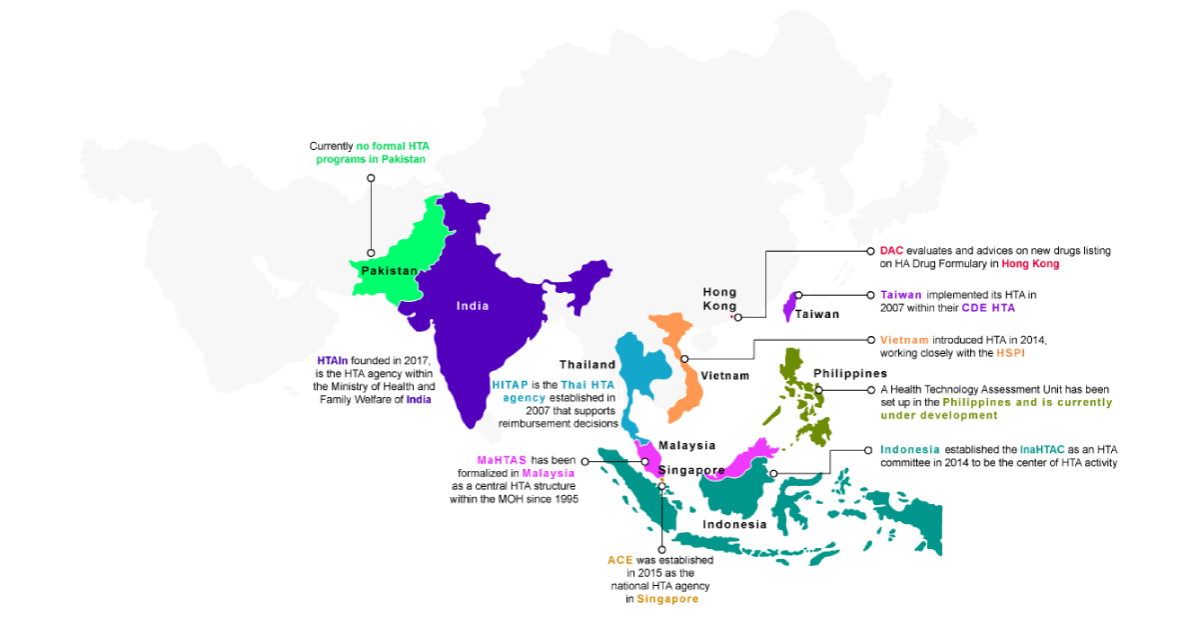
Timeline of HTA development across selected 10 countries in Asia. ACE: Agency for Care Effectiveness; CDE: Centre for Drug Evaluation; DAC: Drug Advisory Committee; HA: Hospital Authority; HITAP: Health Intervention and Technology Assessment Program; HSPI: Health Strategy and Planning Institute; HTA: health technology assessment; HTAIn: Health Technology India; InaHTAC: Indonesian Health Technology and Assessment Committee; MaHTAS: Malaysian Health Technology Assessment; MOH: Ministry of Health; UHC: universal health coverage.

Using these archetypes generated from the 2 scoping reviews, this viewpoint review intends to evaluate evolving trends and patterns in the use of various RWD warehouses and contextualize these findings within the broader health care and economic trends. Studying these evolving trends is crucial as it helps understand how RWD warehouses are generating RWE, which in turn informs health care policy and economic decisions [8]. Health technology assessment (HTA) organizations evaluate the clinical and economic aspects of medicines and health care technologies and recommend reimbursement and other policy criteria. Country HTAs are increasingly incorporating RWE for crucial complementary evidence, particularly in cost-effectiveness analyses [8,9]. However, data accessibility issues, lack of knowledge on robust methodologies, and a shortage of qualified researchers limit the generation of RWE for regulatory and reimbursement decision-making [10]. Our selection of countries for this scoping review was strategically based on the contrasting spectrum of HTA expertise at different timelines of development, from relatively mature systems in Taiwan, Singapore, Hong Kong, Malaysia, and Thailand to emerging frameworks in India, Indonesia, the Philippines, and Vietnam, and nascent systems in Pakistan (Figure 1).

## Country Trends and Key Identified Databases for 10 Asian Countries

Harnessing RWD is pivotal for improving health care outcomes, and understanding country-specific trends and the databases that underpin this research is crucial [11,12]. The following section provides a granular view of the health care and RWD landscape across 10 Asian countries, dissecting the trends in research publications and the key linked databases that have been pivotal in generating RWE (Multimedia Appendix 2 [13-20]). Countries are listed in order of highest to lowest counts of generated RWD publications in the last few years [6,7].

## Solo Scholars

### Taiwan

**Figure 2:**
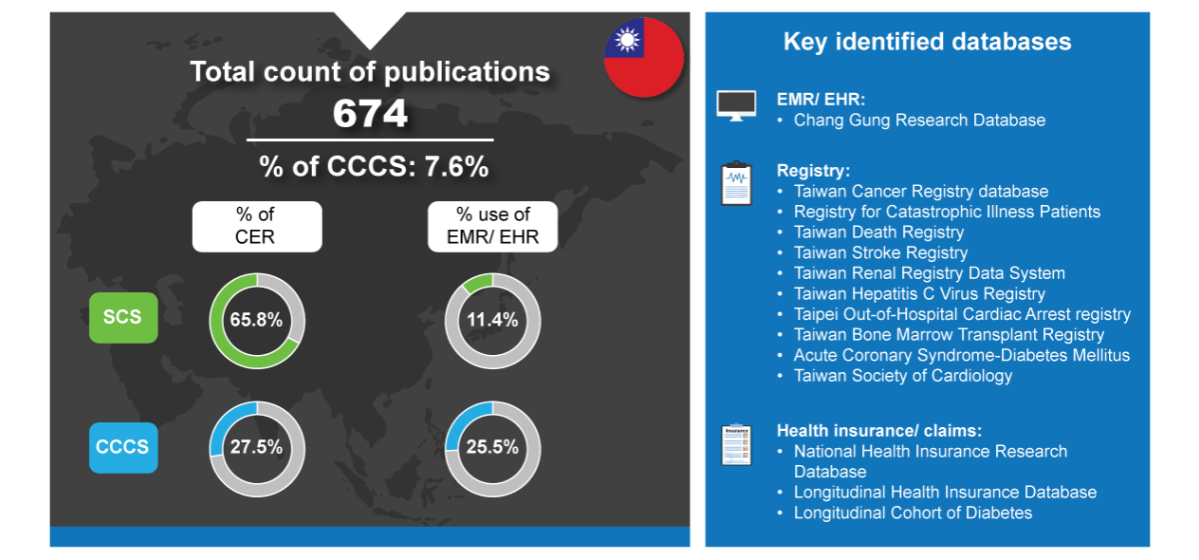
Real-world data landscape for Taiwan (2017-2022). CCCS: cross-country collaboration studies; CER: comparative effectiveness research; EHR: electronic health record; EMR: electronic medical record; SCS: single-country studies.

Taiwan is a thriving democracy with a population of 23.3 million and a prosperous market economy. Taiwan’s gross domestic product (GDP) grew by 2.45% in 2022 [21]. Development of the digital infrastructure in health care is one of the Taiwan government’s major goals in the next few years [22]. Although there seems a lower overall usage of EMRs or electronic health records (EHRs) in SCS for Taiwan (71/623, 11.4%) (Figure 2), there was a noticeable increase in their usage from 2.8% (1/36) to 19.4% (20/103) from 2017 to 2022 [6]. As shown in Figure 2, Taiwan has a high percentage of CER in their SCS (410/623, 65.8%). Inversely, the trend for higher CER is not seen in the CCCS (14/51, 27.5%).

Taiwan has a robust health care system, which is reflected in the percentages describing their RWD publications. Following the creation of the National Health Insurance (NHI) in 1995, Taiwan has set a benchmark in the region with a successful universal health coverage (UHC) scheme in their health care system [23]. Enrollment in the NHI provides coverage for essential medical care and pharmaceuticals for all residents [24]. The success of this system is exemplified in Taiwan’s favorable result on the world’s health and health systems ranking in 2023 [25], on which they were placed fourth (based on the Legatum Prosperity Index that also accounts for economic and social well-being). Furthermore, most health care facilities in Taiwan upload the claims data of each visit to NHI, including patient visits, drug prescriptions, surgeries, and examinations [23]. However, Taiwan’s NHI has changed since its creation in 1995 and continues to evolve [26]. In 2007, Taiwan implemented its HTA within their Centre for Drug Evaluation to evaluate the financial suitability and clinical effectiveness of new drugs for reimbursement decisions [27]. This evolved into the formation of the Division of HTA in 2008 [28]. The high proportion of NHI drug expenses was a huge burden on Taiwan’s health care system, and there were general recommendations to have a reduction in drug expenditure [29]. As such, with this goal of reducing drug expenditures, some behind-the-counter products were delisted from Taiwan’s national Drug Reimbursement Scheme [23]. Along with this, coverage change was aimed at reshaping patient expectations and attitudes toward health. Taiwan’s vision for 2030 includes “precision health,” which places an important focus on health promotion. This concept of precision health involves using data to gather sufficient information that can be used to predict health risks and prevent diseases at the population level [30]. This vision relies on adopting descriptive RWD to produce RWE that can aid in the refinement of specific health policies that contribute to precision health and less comparative research. This also partially explains why there was a decrease in the publication of CER studies most notably from 75% in 2018 to 60% in 2022 [6]. It is worth noting that the literature search on the publication of RWE from linked databases in Taiwan was conducted in September 2022 on PubMed, and the analysis assumed a linear distribution for studies in 2017 and 2022.

Taiwan stands out as a significant contributor to RWD studies in Asia, predominantly through SCS, because it employs a diverse array of databases and holds a wealth of insurance claims data.

The key identified databases contributing to RWD publications included the following [6]:

- *Chang Gung Research Database (CGRD)*: CGRD is a multi-institutional EMR database collected from the Chang Gung Memorial Hospital system, which is the largest medical system in Taiwan [31]. Except for 2 municipal hospitals, all EMR data from Chang Gung Memorial Hospital are included in the CGRD. The database includes approximately 6%-20% outpatient and 10%-12% inpatient claims records [32,33].
- *Taiwan Cancer Registry (TCR) Database*: TCR was developed to monitor cancer incidence at the national level. The TCR holds basic information on patients with newly diagnosed cancer from hospitals with >50 beds throughout the country [34].
- *Registry for Catastrophic Illness Patients*: It is associated with the National Health Insurance Research Database (NHIRD) and is used to identify patients with catastrophic illness in the Taiwan health insurance system [35].
- *Taiwan Death Registry*: It contains information on accurate causes of death and dates for all residents of Taiwan [36].
- *Taiwan Stroke Registry*: This is a nationwide hospital-based registry that enrolls patients with stroke from 19 academic medical centers, 37 regional hospitals, and 8 district hospitals. The data are collected prospectively by trained neurologists and study nurses [37].
- *Taiwan Renal Registry Data System*: It collects patients’ clinical and laboratory information from all dialysis units in Taiwan [38].
- *Taiwan Hepatitis C Virus Registry*: This registry program is a nationwide hepatitis C virus platform implemented by the Taiwan Association for the Study of the Liver [39].
- *Taipei Out-of-Hospital Cardiac Arrest Registry*: It includes prehospital and hospital information on patients with out-of-hospital cardiac arrest in Taipei [40].
- *Taiwan Bone Marrow Transplant Registry*: This registry holds clinical data from consecutive allogeneic hematopoietic cell transplant recipients from 15 transplant centers in Taiwan [41].
- *Acute Coronary Syndrome-Diabetes Mellitus Registry*: It is a nationwide registry of patients with acute coronary syndrome in Taiwan that collects real-world clinical practices and outcomes data [42].
- *Taiwan Society of Cardiology Registry*: This registry collects data from patients at 21 medical centers or teaching hospitals in Taiwan from patients who are hospitalized with acute new-onset heart failure or acute decompensated chronic heart failure with a reduced ejection fraction [43].
- *NHIRD*: It covers >99.6% of the Taiwanese population and collects claims data from outpatient and inpatient hospital care settings [26].
- *Longitudinal Health Insurance Database*: This database is derived from the NHI system and includes the registration files and original reimbursement claims of a million randomly selected beneficiaries, under the NHI program [44].
- *Longitudinal Cohort of Diabetes*: This is a de-identified subset of data from the NHIRD [45].

### Singapore

**Figure 3:**
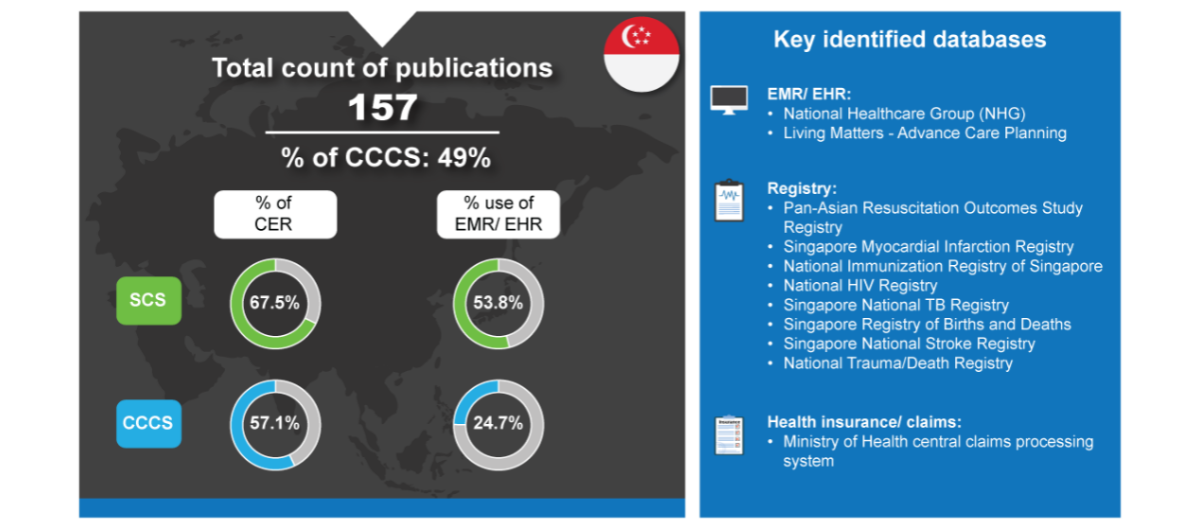
Real-world data landscape for Singapore (2018-2023). CCCS: cross-country collaboration studies; CER: comparative effectiveness research; EHR: electronic health record; EMR: electronic medical record; SCS: single-country studies; TB: tuberculosis.

Singapore is a prosperous nation with one of the highest GDP per capita in Asia. In 2022, Singapore’s GDP grew by 3.6% [46]. In 2023, the Singapore government announced the plan to spend US $2.5 billion on info-communications technology with the redevelopment of major hospitals [47]. Compared with Taiwan, Singapore relies on higher use of EMRs or EHRs for real-world SCS (43/80, 53.8%), which is balanced with their use of clinical registries (46/80, 57.5%) [7]. Although Singapore SCS was predominantly in CER style (54/80, 67.5%), unlike Taiwan, the percentage of CCCS publications remained relatively high (57/77, 57.1%; Figure 3).

In 2023, Singapore was regarded as top-ranking in the world’s health and health systems, 3 places ahead of Taiwan [25]. Despite emerging issues surrounding health care services and the affordability of health care, Singapore is able to achieve good health outcomes with a health index score of 86.9 through a hybrid public and private health care model that is dominated by public hospitals in the hospital sector and private clinics in the outpatient sector [25,48]. Trusted Research and Real World Data Utilization or TRUST platform stands as the cornerstone for national data exchange in Singapore by facilitating the secure and anonymized sharing of health-related RWD between public and private health care sectors [49]. The Ministry of Health (MOH) aimed to increase health care capacity to enhance health care affordability in the Healthcare 2020 Masterplan [50]. Within the MOH, the Agency for Care Effectiveness was established in 2015 as the national HTA agency with the aim of evaluating drugs for subsidization and medical technologies [51,52]. This may partially explain Singapore’s relatively high percentage of CER publications in both SCS and CCCS, particularly as these types of studies enable a comparison of different medications and health management strategies. This is further magnified by the observation that the percentage of SCS CER publications from Singapore increased from 2018 to 2023 (analysis of these publications assumed a linear distribution for studies in 2018 and 2023) [7].

The key identified databases contributing to RWD publications from Singapore included the following [7]:

- *National Healthcare Group*: This record links key administrative and clinical information from a group of National Healthcare Group health care institutions for patients with chronic diseases such as diabetes mellitus, hypertension, dyslipidemia, stroke, cardiovascular diseases, and chronic renal disease [53].
- *Living Matters—Advance Care Planning (ACP)*: This framework provides a comprehensive web-based resource about ACP, including options for documenting patients’ care preferences, particularly valuable in emergencies or when making critical care decisions. ACP details are also integrated into EMR or EHR, and all health care providers involved in patient care can easily access and understand a patient’s end-of-life care preferences [54].
- *Pan-Asian Resuscitation Outcomes Study Registry*: The Pan-Asian Resuscitation Outcomes Study registry contains information from dispatch centers, ambulances, and hospitals from 7 countries in the Asia Pacific region (Japan, South Korea, Taiwan, Thailand, United Arab Emirates-Dubai, Singapore, and Malaysia) [55].
- *Singapore Myocardial Infarction Registry*: It is an island-wide registry that is being managed by the National Registry of Diseases Office that contains epidemiological data on acute myocardial infarction cases diagnosed in public and private sector hospitals and some out-of-hospital deaths certified by medical practitioners in Singapore [56].
- *National Immunization Registry of Singapore*: This registry collects and maintains accurate, complete, and current vaccination records of children and adults living in Singapore [57].
- *National Human Immunodeficiency Virus Registry*: This registry is a name‐based system that holds identifiable data for known human immunodeficiency virus cases in Singapore, as well as contacts of cases that were reported [58].
- *Singapore National Tuberculosis Registry*: This registry is electronically linked to the 2 mycobacterial culture laboratories in Singapore and captures all positive Mycobacterium tuberculosis complex culture results in the country [59].
- *Singapore Registry of Births and Deaths*: This death registry is maintained by the Ministry of Home Affairs Immigration and Checkpoints Authority. It collects data on the cause and date of death of all Singaporeans and permanent residents in Singapore [60].
- *Singapore National Stroke Registry*: This is a countrywide registry of risk factors, stroke subtypes, management, and outcomes of incident and recurrent stroke in Singapore [61].
- *National Trauma/Death Registry*: This is a registry that contains anatomical injury codes, indicators of physiological response to injury, and patient demographics [62].
- *MOH’s Central Claims Processing System*: It is used in Singapore to process the patient’s MediSave and MediShield claims [63].

### India

India is the largest lower-middle-income country in the world and accounts for around 18% of the total population [64]. According to Nexdigm, the Indian health care industry is expected to reach more than US $610 billion by 2026, as there is a growing demand for specialized and higher-quality health care facilities [65]. Hospitals, clinical trials, telemedicine, medical tourism, medical devices, medical and diagnostic equipment, and health insurance are among the key products and services that would drive this growth [66].

**Figure 4:**
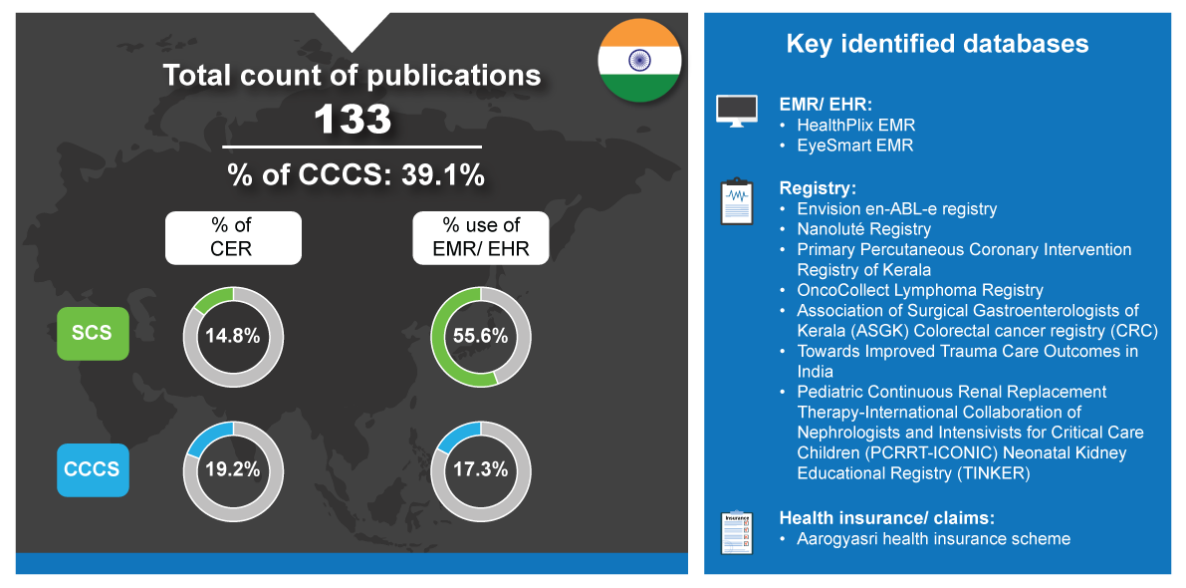
Real-world data landscape for India (2017-2022). CCCS: cross-country collaboration studies; CER: comparative effectiveness research; EHR: electronic health record; EMR: electronic medical record; SCS: single-country studies.

Compared with Taiwan and Singapore, India has a less extensive health care system [25], which is also reflected in its comparatively lower percentage of SCS (12/81, 14.8%) and CCCS (10/52, 19.2%; Figure 4). India attracts a growing medical tourism market with a growth of 22%-25% from 2014 and advocacy for adoption of EMR across the country [67]. Among RWD databases, the studies primarily use EHR or EMR (45/81, 55.6%), with an increasing trend from 20% in 2017 to 48% in 2022 [6]. Its usage is expected to continue to rise with the National Digital Health Mission, since 2020 [68]. India has made commitments toward achieving UHC and has been producing policies and institutional changes that are directed toward increasing health coverage and access to health services [69]. Government-funded health care sector is the provider of health care to lower-income populations; however, the private health care sector is the dominant health care provider [70]. In 2020, 70% of hospital market share was controlled by private sector providers, and 63% of hospital beds were provided by the hospital sector [71,72].

India launched Ayushman Bharat—one of the most ambitious health missions ever to achieve UHC in 2018 [73]. Ayushman Bharat encompasses 2 complementary schemes, Health and Wellness Centres and the National Health Protection Scheme [69]. Along with this, there have been other initiatives to achieve UHC [69]. The Pradhan Mantri Jan Arogya Yojana scheme provides secondary and tertiary hospital care insurance to the bottom 40% of the population [68]. Previously called the “Medical Technology Assessment Board,” HTAIn, founded in 2017, is the HTA agency within the Ministry of Health and Family Welfare tasked with developing an HTA system to aid in decision-making for resource allocation at the national and state levels [74,75]. Reflecting these policy aims, the proportion of CER for SCS and CCCS was low in India but increased from 2017 to 2022 (12/81, 14.8% and 10/52, 19.2%, respectively, Figure 4), assuming a linear distribution for studies in 2017 and 2022 [6].

The key identified databases contributing to RWD publications from India included the following [6]:

- *HealthPlix EMR*: This is an artificial intelligence–powered electronic medical software system in India [76].
- *EyeSmart EMR*: This is an EMR and Hospital Management System in India that integrates the clinical, surgical, financial, and operational functions of the LV Prasad Eye Institute on a single platform [77].
- *Envision en-ABL-e Registry*: This was a multicenter registry that enrolled 2500 patients treated with 3286 Abluminus DES (Envision Scientific, Surat, India) across 31 centers across the country from June 2012 to December 2018 [78].
- *Nanoluté Registry*: Nanoluté registry was used to observe the clinical performance of a novel sirolimus-coated balloon (Concept Medical Research Private Limited, India) for treating coronary de novo and restenotic lesions [79].
- *Primary Percutaneous Coronary Intervention Registry of Kerala*: This registry is a large multicenter primary percutaneous coronary intervention registry from Kerala, India. It reports long‐term outcomes of patients presenting with ST‐segment–elevation myocardial infarction to percutaneous coronary intervention–capable hospitals or facilities [80].
- *OncoCollect Lymphoma Registry*: This registry was set up in 2017 as a collaborative group effort to evaluate current practices for managing diffuse large B-cell lymphoma in middle-income countries [81].
- *Association of Surgical Gastroenterologists of Kerala Colorectal Cancer Registry*: This registry collects demographics and perioperative outcomes of colorectal cancer in Kerala from volunteer members of the Association of Surgical Gastroenterologists of Kerala [82].
- *Towards Improved Trauma Care Outcomes in India*: It is a multicenter trauma registry that contains data on trauma patients admitted to 4 public university hospitals in Mumbai, Delhi, and Kolkata from October 1, 2013, to September 30, 2015 [83].
- *The Indian Pediatric Continuous Renal Replacement Therapy-International Collaboration of Nephrologists and Intensivists for Critical Care Children Neonatal Kidney Educational Registry*: This registry is a database of all admitted neonates ≤28 days who received intravenous fluids for at least 48 hours [84].
- *Aarogyasri Health Insurance Scheme*: This is a social insurance scheme with a private-public partnership model to deal with the problems of medical expenditures for poor households [85].

### Hong Kong

Health care services in Hong Kong are provided by both private and government-funded public sectors, with public medical services provided by the Department of Health and the Hong Kong Hospital Authority (HA) [86]. Hong Kong has a well-developed health care system that ranked 14th among the health and health systems ranking of countries worldwide in 2023 [25]. Hong Kong’s system is a parallel, segmented public and private financed and provided health care, where the public sector accounted for 51% of total health expenditure and the private sector accounted for 49% in 2017/18 [87]. With the government’s commitment to provide UHC, the Drug Advisory Committee is used to evaluate and advise on new drugs listed on the Hospital Authority Drug Formulary, which is the largest public health care service provider in Hong Kong. One of the missions of the Drug Advisory Committee is to ensure equal access of patients to cost-effective drugs with proven safety and efficacy [88].

**Figure 5:**
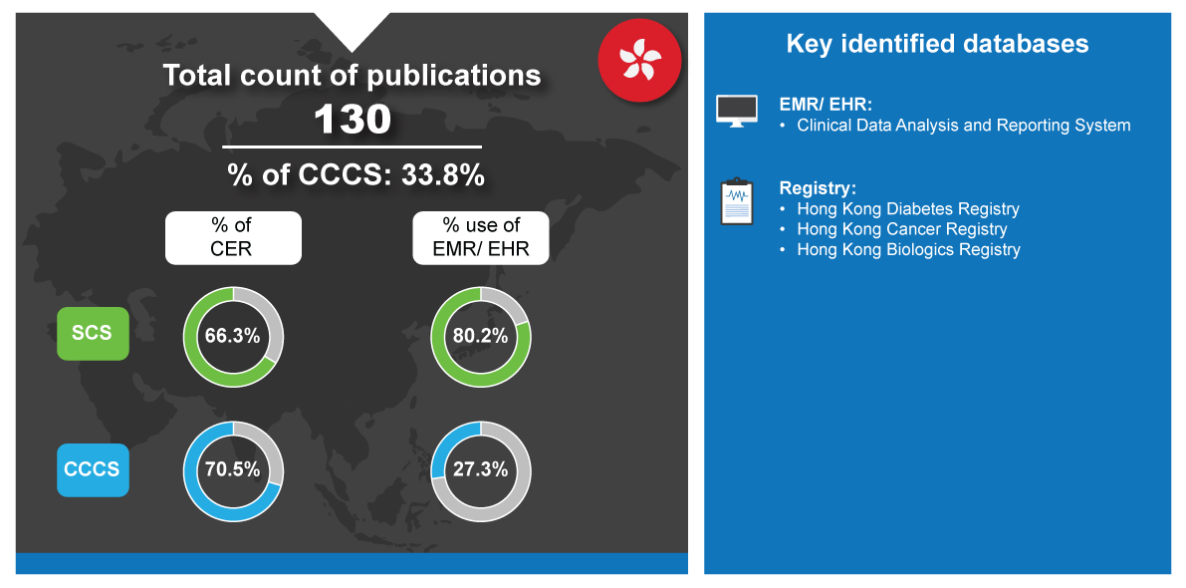
Real-world data landscape for Hong Kong (2018-2023). CCCS: cross-country collaboration studies; CER: comparative effectiveness research; EHR: electronic health record; EMR: electronic medical record; SCS: single-country studies.

As shown in Figure 5, CER publications made up 66.3% (57/86) of SCS publications and 70.5% (31/44) of CCCS. To put these high percentages into context, the drug appraisal and review process involves an assessment of clinical evidence and health economic evidence that is then used to make recommendations on reimbursement of new drugs [89]. In Hong Kong, there has been an increase in the requirement for a systematic HTA, placing emphasis on the value and comparison of emerging and conventional drugs [88]. In line with this, there is an upward trend in the percentage of SCS CER publications for Hong Kong from 2018 to 2023, assuming a linear distribution for studies in 2018 and 2023 [7].

The key identified databases contributing to RWD publications from Hong Kong included the following [7]:

- *Clinical Data Analysis and Reporting System (CDARS)*: CDARS is a centralized database developed for research and audit purposes. It is managed by the HA. It collects anonymized records of demographics, admission, prescription, diagnosis, procedure, laboratory test, and death information [90].
- *Hong Kong Diabetes Registry*: This is a diabetes registry used for quality assurance and risk stratification to facilitate subsequent triage of patients to different clinic settings [91].
- *Hong Kong Cancer Registry*: This is a population-based registry committed to collecting and conducting analyses on data from all cancer cases in Hong Kong [92].
- *Hong Kong Biologics Registry*: This is a registry that was established in December 2005 by The Hong Kong Society of Rheumatology to capture efficacy and safety data regarding the use of biological agents for the treatment of rheumatic disease [93].

### Malaysia

Malaysia is an upper-middle-income country with a population of about 34 million [94]. It is located at the heart of Southeast Asia and represents one of the regional hubs for information and communication technology and medical travel [95]. Digitalization of information forms across major industrial sectors will help secure Malaysia’s role in the future global economy. As of 2023, Malaysia was ranked 42nd on the health and health systems of countries worldwide [25]. Malaysia’s health care system consists of tax-funded and highly subsidized government-led services, with a fast-growing private sector [96]. Their dual-tier system consists of a government-led public sector with an existing private sector, and in 2019, their public health expenditure amounted to 52% of their total health expenditure.

**Figure 6:**
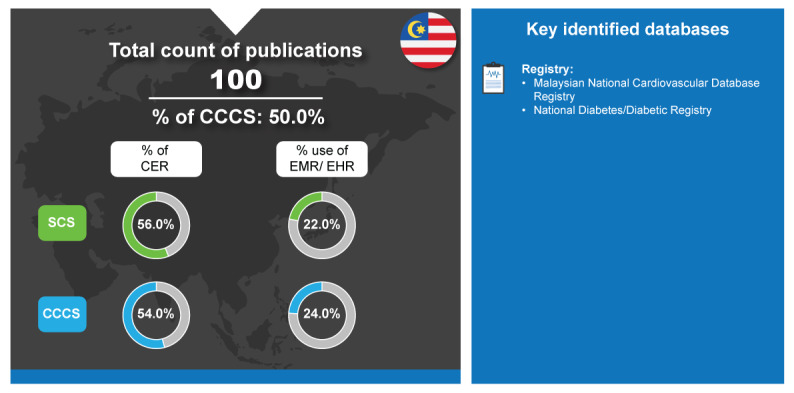
Real-world data landscape for Malaysia (2018-2023). CCCS: cross-country collaboration studies; CER: comparative effectiveness research; EHR: electronic health record; EMR: electronic medical record; SCS: single-country studies.

Malaysia’s 2023 Health White Paper outlines a transformative masterplan for health management information and data system [97]. They aim to improve the health outcomes and well-being of Malaysians and the use of the Malaysian Health Technology Assessment, also termed as MaHTAS. HTA has been formalized in Malaysia as a central structure within the MOH since 1995 and is a trusted medical evidence source [97]. Their generation of RWD publication used registries, with no, or low contributions from other database types [7] (Figure 6). In line with other countries that use HTA, 56% (28/50) of SCS were CER, as were 54% (27/50) of the CCCS [7].

The key identified databases contributing to RWD publications from Malaysia included the following [7]:

- *Malaysian National Cardiovascular Database Registry*: This is a service supported by the Malaysian MOH to collect information about cardiovascular diseases [98].
- *National Diabetes/Diabetic Registry*: This is a registry used to enable tracking of glycemic control and clinical outcomes of patients with diabetes managed at MOH health clinics [99].

### Thailand

Thailand is also an upper-middle-income country but with the second largest economy (after Indonesia) in the Association of Southeast Asian Nations (ASEAN). Its GDP in 2022 was US $526 billion [100]. Thailand has the fourth greatest number of Joint Commission International accreditation–certified hospitals after Saudi Arabia, the United Arab Emirates, and Brazil. Yet the medical expenses in Thailand are 50%-80% lower than those in Europe, the United States, and Canada [101].

**Figure 7:**
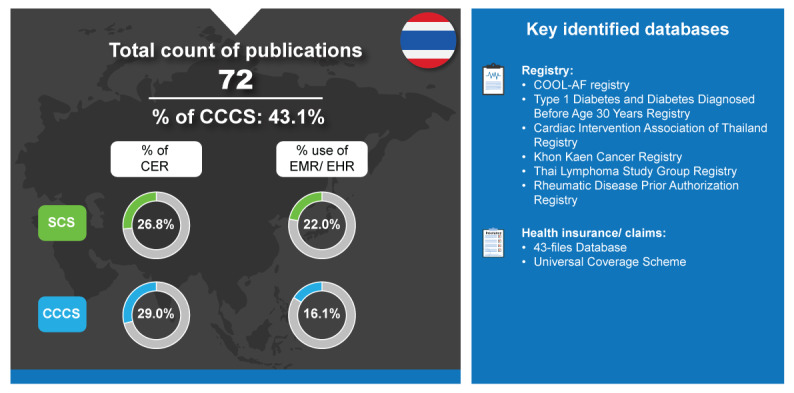
Real-world data landscape for Thailand (2017-2022). CCCS: cross-country collaboration studies; CER: comparative effectiveness research; COOL-AF: Cohort of Antithrombotic Use and Optimal International Normalized Ratio Levels in Patients with Atrial Fibrillation; EHR: electronic health record; EMR: electronic medical record; SCS: single-country studies.

On the health and health systems ranking of countries worldwide in 2023, Thailand was ranked 13th [25]. In 2020, the private health expenditure in Thailand accounted for about 71.2% of the total health expenditure [102]. Furthermore, Thailand has become internationally known for its success with UHC policy since its development in 2002 [103]. The National List of Essential Medicines is a drug reimbursement list for the public health insurance schemes in Thailand, and the Health Intervention and Technology Assessment Program is a Thai HTA agency established in 2007 that supports reimbursement decisions [104]. Thailand used a relatively low percentage of EHR or EMR warehouses in their RWD publications, with only 22% contribution in SCS publications and 16.1% in CCCS publications (Figure 7). Surprisingly, they also had a relatively low percentage of CER publications with 26.5% of their SCS being CER publications and 29% of the CCCS being CER publications [6].

The key identified databases contributing to RWD publications from Thailand included the following [6]:

- *COOL-AF Registry*: The “Cohort of Antithrombotic Use and Optimal International Normalized Ratio Levels in Patients with Atrial Fibrillation (COOL-AF)” registry is a database of patients with atrial fibrillation in Thailand [105].
- *Type 1 Diabetes and Diabetes Diagnosed Before Age 30 Years Registry*: This registry was established in 2014 and involves 31 hospitals to evaluate glycemic control and complications in patients with type 1 diabetes [106].
- *Cardiac Intervention Association of Thailand Registry*: This nationwide registry was an initiative of the Cardiac Intervention Association of Thailand. All cardiac catheterization laboratories in Thailand were invited to participate [107].
- *Khon Kaen Cancer Registry*: This is a population-based cancer registry of Khon Kaen that covers 26 districts in Northeastern Thailand [108].
- *Thai Lymphoma Study Group Registry*: This is a web-based nationwide lymphoma registry from the Thai Lymphoma Study Group [109].
- *Rheumatic Disease Prior Authorization Registry*: This is a national registry used for government reimbursement in the Rheumatic Disease Prior Authorization system. This registry contains data on patients’ demographic and clinical characteristics at baseline, data-related disease activity, and type of biologic medication prescribed [110].
- *43-Files Database*: These are administrative data collected by the Thai Ministry of Public Health for the purpose of reimbursement and health service use [111].
- *Universal Coverage Scheme*: This health coverage scheme consists of 3 main public insurance schemes that offer full-service coverage. The 3 schemes are the Civil Servants Medical Benefits Scheme for civil servants and their dependents, Social Health Insurance for private sector employees, and the Universal Coverage Scheme that covers 70% of the population in Thailand [112,113].

## Global Collaborators

### Indonesia

**Figure 8:**
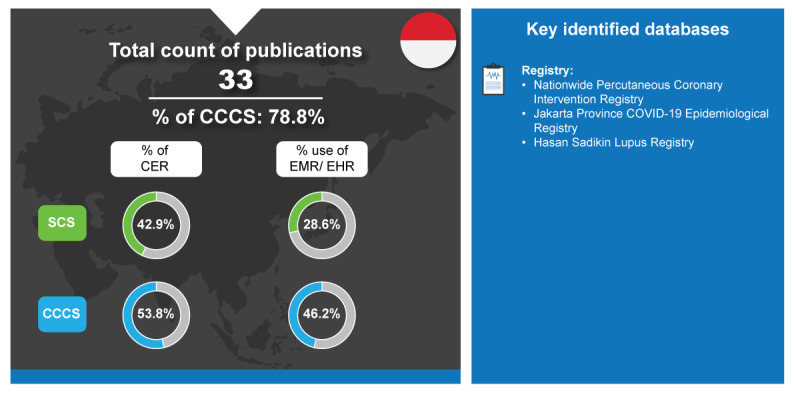
Real-world data landscape for Indonesia (2018-2023). CCCS: cross-country collaboration studies; CER: comparative effectiveness research; EHR: electronic health record; EMR: electronic medical record; SCS: single-country studies.

Indonesia is a country of 279.5 million people and is South East Asia’s largest economy with a GDP of ~US $1.32 trillion in 2022. Ranked 97th in the 2023 health and systems ranking, Indonesia is progressing their UHC through the expansion of NHI (Jaminan Kesehatan Nasional scheme) [25,114,115]. The Indonesian government established the Indonesian Health Technology and Assessment Committee as an HTA committee in 2014 to be the center of HTA activity, and as a starting point for the development of HTA [116]. Similar to the other Solo Scholars in this study, Indonesia was responsible for a relatively low number of RWD publications [7]. Of their SCS, 28.6% (2/7) used EMR and 42.9% (3/7) were CER (Figure 8).

The key identified databases contributing to RWD publications from Indonesia included the following [7]:

- *Nationwide Percutaneous Coronary Intervention Registry*: This is a multicenter registry of interventional cardiology projects involving 9 centers across Indonesia [117].
- *Jakarta Province COVID-19 Epidemiological Registry*: This is a database of patients with confirmed COVID-19 cases from Jakarta province [118].
- *Hasan Sadikin Lupus Registry*: This is a registry created in January 2016 that reports the medical records of patients with systemic lupus erythematosus from the Dr Hasan Sadikin General Hospital [119].

### Pakistan

Health care has been identified as one of the best industry prospects for Pakistan, a country with a population of more than 240 million and a GDP growth rate of 0.29% in 2023 [120]. Ranked 124th on the health and health systems ranking of countries in 2023, Pakistan’s health care comprises a 3-tier system of primary, secondary, and tertiary levels. The public and private sectors work together to provide the best possible care, but there have been tremendous concerns about the failure of the delivery of quality care due to various factors, ranging from inadequate infrastructure to inequitable distribution of health care facilities [121]. Of note, to our knowledge, there are currently no formal HTA programs in Pakistan [122].

**Figure 9:**
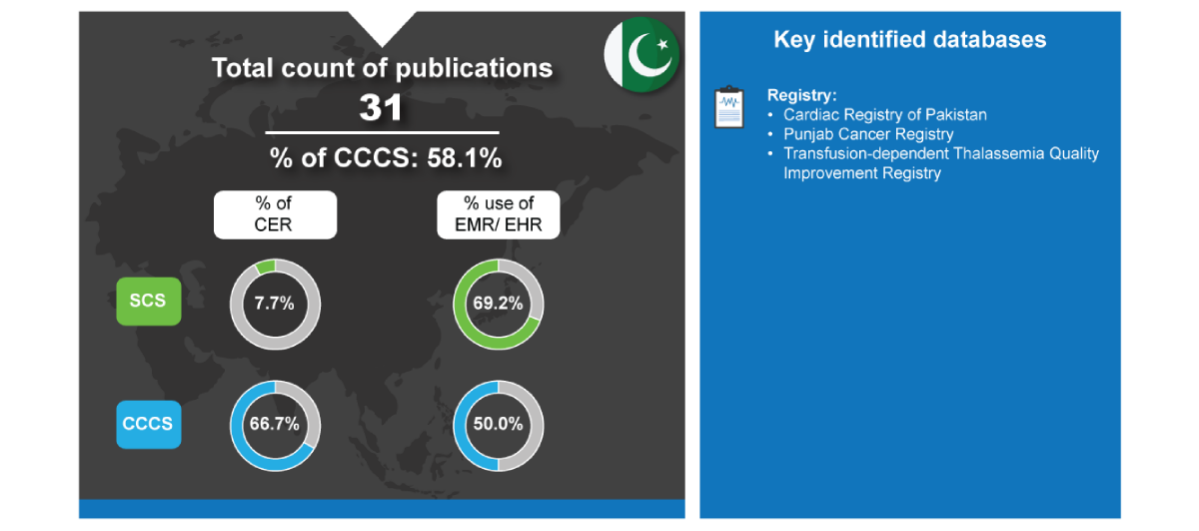
Real-world data landscape for Pakistan (2018-2023). CCCS: cross-country collaboration studies; CER: comparative effectiveness research; EHR: electronic health record; EMR: electronic medical record; SCS: single-country studies.

These challenges are reflected in their low number of publications and, more specifically, their low number of SCS. Among the SCS, only 7.7% (1/13) were CER studies. Alternatively, due to their inadequate infrastructure, more CER was conducted in CCCS (12/18, 66.7%) (Figure 9).

The key identified databases contributing to RWD publications from Pakistan included the following [7]:

- *Cardiac Registry of Pakistan*: This is a cardiac registry that includes 25 facilities from 3 provinces of Pakistan. Standardized data are collected every 3 weeks for this database [123].
- *Punjab Cancer Registry*: This registry collects population-level cancer statistics in Pakistan [124].
- *Transfusion-Dependent Thalassemia Quality Improvement Registry*: It is a database comprising patients with transfusion-dependent thalassemia from 4 centers in Karachi, Pakistan [125].

### Vietnam

In the 2022 ASEAN Business Outlook Survey, AmCham Singapore members indicated Vietnam as the top Asia Pacific country (30%), where companies are considering expansion, followed by Malaysia (25%), Thailand (24%), and Indonesia (23%) [126]. The per capita GDP for Vietnam was US $4086 in 2022 and is meant to increase to at least US $18,000 by 2035. A large population of almost 100 million (half of which are younger than 30 years), consistent strong economic growth, and ongoing reform have created a dynamic and rapidly evolving commercial environment in Vietnam [126].

Vietnam was ranked 44th on the health and health systems ranking of countries in 2023 [25]. The Vietnamese MOH manages 3 levels of health service delivery: primary level in districts and communes, secondary level in provinces, and tertiary level in national institutions under central government control. Working within this hierarchical system, Vietnam aims to build a solid health care infrastructure from the grassroots [127]. Despite the significant progress, the health care system still faces challenges to keeping ahead of the country’s escalating population [127]. Vietnam is making significant progress toward achieving UHC and is committed to delivering UHC [127]. In 2014, Vietnam introduced the HTA, working closely with the Health Strategy and Planning Institute to facilitate its institutionalization [128].

**Figure 10:**
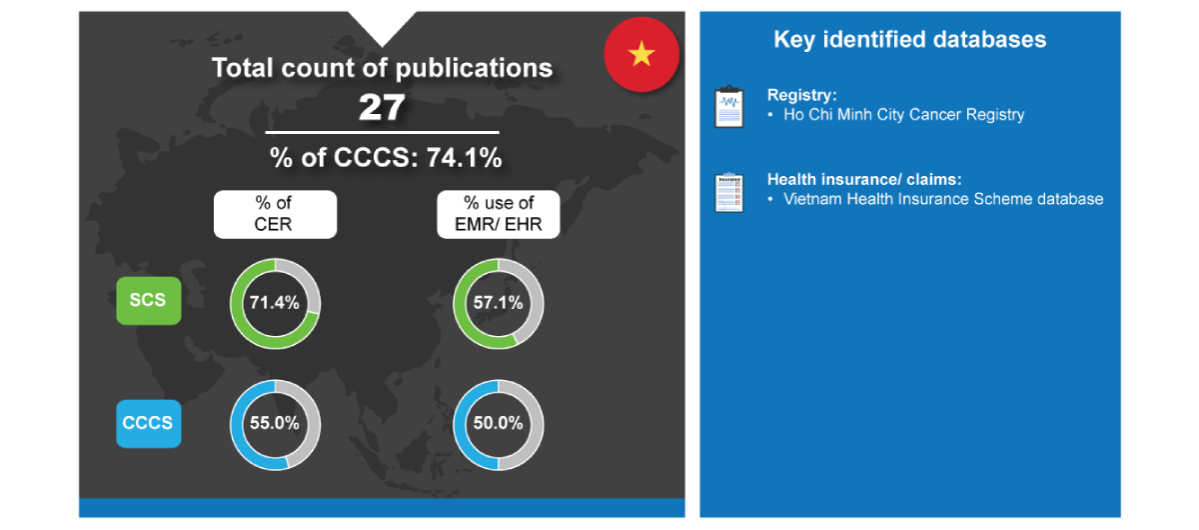
Real-world data landscape for Vietnam (2018-2023). CCCS: cross-country collaboration studies; CER: comparative effectiveness research; EHR: electronic health record; EMR: electronic medical record; SCS: single-country studies.

Similar to the other Global Collaborators, Vietnam generated a low percentage of publications. However, 71.4% (5/7) of SCS and 55% (11/20) of CCCS were CER (Figure 10). Based on the literature search of RWE publications from linked databases in Vietnam conducted in May 2023 on PubMed where the analysis assumed a linear distribution for studies in 2019 and 2023, Vietnam also exhibited the most significant increase with a growth rate of 24.5% in RWD publications in the last 5 years [7].

The key identified databases contributing to RWD publications from Vietnam included the following [7]:

- *Ho Chi Minh City Cancer Registry*: This registry documents all diagnosed cancer cases in Ho Chi Minh City [129].
- *Vietnam Health Insurance Scheme Database*: This claims database is managed by the Vietnam Social Security Service and it contains medical examinations and care, preventive care, rehabilitation, maternity care, and prescribed medications [130].

### Philippines

**Figure 11:**
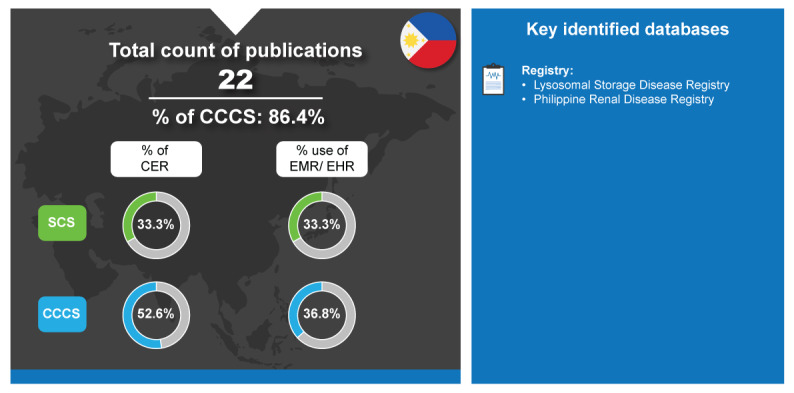
Real-world data landscape for Philippines (2018-2023). CCCS: cross-country collaboration studies; CER: comparative effectiveness research; EHR: electronic health record; EMR: electronic medical record; SCS: single-country studies.

The health care system is shared between the public and private sectors in Philippines and was ranked 96th on the health and health systems ranking of countries worldwide in 2023 [25]. To respond to health financing gaps, the Philippines made major reforms including the passage of the Universal Health Care Law in 2019; however, there are ongoing issues with expenditure [131]. Following this passage, there were increased efforts to institutionalize HTA in the Philippines and as a result, a health technology assessment unit was set up and is under development [52]. During the time period of 2018-2023, the majority of the Philippines’ RWD publications were CCCS (19/22, 86.4%) and only 33.3% (1/3) each of SCS were CER and used EMR (Figure 11). Although the adoption of EMR for research is low and the publication count has been scanty in the last 5 years [7], the Philippines is estimated to spend approximately US $4.4 billion on digital infrastructure over the next 6 years [132].

The key identified databases contributing to RWD publications from the Philippines included the following [7]:

- *Lysosomal Storage Disease Registry*: It is a registry of Filipinos diagnosed with any lysosomal storage disease [133].
- *Philippine Renal Disease Registry*: This is a registry consisting of 2 major components: The Chronic Renal Disease Registry and the End Stage Renal Disease Registry. The Chronic Renal Disease Registry is composed of the Renal Biopsy Registry, and the End Stage Renal Disease Registry is composed of Haemodialysis, Peritoneal Dialysis, and Transplant Registries [134].

## Discussion

RWD is used to inform policies in different capacities across the countries. Focusing on the types and sources of RWD, we noted key differences across the countries. For example, Taiwan has a strong market economy [21], with a robust and successful health care system [25]. It was noted that they had fewer publications using EHR or EMR and more so used health insurance or administrative claims for their numerous publications. They also had many identified databases and, as such, were able to access several data sources to generate RWE (Figure 1). Taiwan exemplifies a country with the infrastructure and resources to comfortably produce RWE. A trend in Solo Scholar countries such as Taiwan was that they are prolific in the publication of SCS, with a general decrease in CER studies over time [6,7]. Typically, Global Collaborators have less robust economies and health care systems. These were countries that had less variety in data sources within their health system and a lack of standardized or integrated databases. These countries published more CCCS with a general increasing trend in the percentage of CER studies [7].

For Global Collaborators, CCCS are important for the generation of RWE. This highlights the necessity for data-sharing strategies and a need for the development of data-sharing frameworks, particularly to aid countries that have weaker health care systems and less established infrastructure for RWD [7]. It also underscores the potential benefits of opening data initiatives, where governments play a pivotal role [135]. Opening these data resources to a broader spectrum of stakeholders, including academic institutions, can spur innovation, improve public health outcomes, and foster a more collaborative ecosystem [136]. Such initiatives not only democratize access to valuable health data but also pave the way for a more inclusive approach to tackling global health challenges. This reflects a growing recognition of the importance of transparency and collaboration between the public sector and external entities in enhancing health care delivery and research [137]. Indeed, many nations such as Vietnam leverage regional and global studies conducted in a CER style to match their requirements for HTA. HTA agencies across Asia could contribute to these types of developments as this may be beneficial to them. In 2011, the HTAsiaLink Network was set up as a collaborative network for HTA agencies in Asia [138]. There is also the International Network of Agencies for Health Technology Assessment, which is an international HTA community, though to our knowledge, Taiwan, Singapore, and Malaysia are the only countries discussed that are part of this network [139]. Cross-sectoral partnerships through HTAsiaLink Network and International Network of Agencies for Health Technology Assessment could help address challenges related to RWE generation, which are much more common in lesser developed countries, and support country HTA and regulatory decision makers in decision-making [140,141]. However, there is also a need to improve database identification and integration across countries as there is no consistent naming of the databases, resulting in difficulty in identifying and analyzing RWD publications across Asia. Outside of reimbursement decisions, these improvements would be helpful for a range of groups including health professionals and researchers, health system managers, researchers, policy makers, and investors. Along with these improvements, there is a need for focused use of EMR to streamline the process of generating RWD for disease surveillance and management [142].

In recent years, an increasing number of Asian countries have begun to recognize the importance of HTA in making informed pricing and reimbursement decisions for health care technologies [143]. This shift toward evidence-based health care policy is critical to ensure that decisions around health care technologies are transparent and equitable [144]. To further enhance the transparency and effectiveness of the HTA process, it is crucial to involve all stakeholders at every step of the implementation. In this context, global observational networks such as the Observational Health Data Sciences and Informatics (OHDSI) program offer a promising avenue for enriching HTA processes with robust RWE. Countries such as Taiwan, Singapore, and India already participate in OHDSI, although the global presence remains limited [145]. By aligning governmental initiatives with the Observational Medical Outcomes Partnership Common Data Model used by OHDSI, there is a unique opportunity to rapidly scale up the generation and use of RWE [146]. Such collaboration broadens the evidence base for HTA and facilitates international cooperation and benchmarking in health care technology assessment. Moreover, linking HTA processes with Observational and Medical Outcomes Partnerships or OHDSI could serve as a catalyst for developing sophisticated and evidence-driven health care policies.

In the Hong Kong government’s 2021-2022 budget, an increase of US $480 million was allotted to the HA to meet increasing demand for health care services [86]. One such service is the CDARS, which emerged as the key EMR database for Hong Kong [7]. It is a database developed by HA, which is readily used in Hong Kong for RWD [90]. Among all 10 countries, Hong Kong leveraged EMR for most of its RWD SCS (80.2%) (Figure 4). Streamlined use of EMRs has the potential to collect a variety of RWD, which reduces the need to set up multiple disease-specific clinical registries for generating non–cost-related RWE. The CDARS database is a good example of this as it has been established for research purposes and contains necessary medical information, including patient demographics, details on admission, diagnosis, and prescription and laboratory tests [90].

### Conclusions

RWD plays a significant role in informing policy decisions across Asia. There are differing trends and patterns in the use of databases for published RWD across Asia and clear gaps in usage of warehouses across countries. However, based on the economic and health care development trends, there seems a great potential in generating fit-for-purpose RWE across distinct health care systems. Global Collaborators demonstrate a reliance on international partnerships for CCCS, due to a strategic drive to overcome infrastructural limitations. The review stresses the necessity for enhanced data-sharing strategies and more robust database integration, which are critical for countries with limited health care system resources. Furthermore, the consistent naming and use of databases, especially EMRs, are pivotal for advancing disease surveillance and RWD generation across Asia. The findings call for joint efforts by HTA agencies and stakeholders to fortify RWD frameworks, which would not only aid reimbursement decisions but also support the broader spectrum of health care stakeholders.

## Appendix

Multimedia Appendix 1 Methodology for plotting Solo Scholars and Global Collaborators archetypes.

Multimedia Appendix 2 Database types used in real-world studies across 10 target Asian countries.

## Abbreviations

ACP: advance care planning
ASEAN: Association of Southeast Asian Nations
CCCS: cross-country collaboration studies
CDARS: clinical data analysis and reporting system
CER: comparative effectiveness research
CGRD: Chang Gung Research Database
COOL-AF: Cohort of Antithrombotic Use and Optimal International Normalized Ratio Levels in Patients with Atrial Fibrillation
EHR: electronic health record
EMR: electronic medical record
GDP: gross domestic product
HA: Hospital Authority
HTA: health technology assessment
MOH: Ministry of Health
NHI: National Health Insurance
NHIRD: National Health Insurance Research Database
OHDSI: Observational Health Data Sciences and Informatics
RWD: real-world data
RWE: real-world evidence
SCS: single-country studies
TCR: Taiwan Cancer Registry
UHC: universal health coverage

## Data Availability

Data generated or analyzed during this study are included in Multimedia Appendices 1 and 2.
