# Supplementary material for: Bridging Real-World Data Gaps: Connecting Dots Across 10 Asian Countries
Source: JMIR Med Inform. 2024 Aug 15;12:e58548. doi: 10.2196/58548 (PMC11362708; doi:10.2196/58548)
Supplement: Multimedia Appendix 1 [file medinform_v12i1e58548_app1.pdf]

Multimedia Appendix 1

Bridging Real-World Data Gaps: Connecting Dots Across 10 Asian Countries

Guilherme Silva Julian , Wen-Yi Shau, Hsu-Wen Chou, Sajita Setia

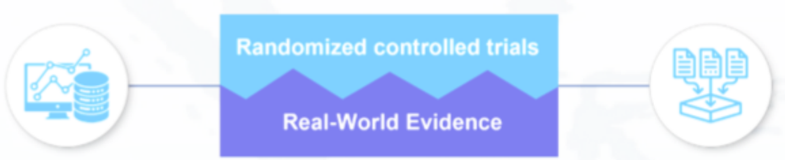

Global Collaborators and Solo Scholars archetypes

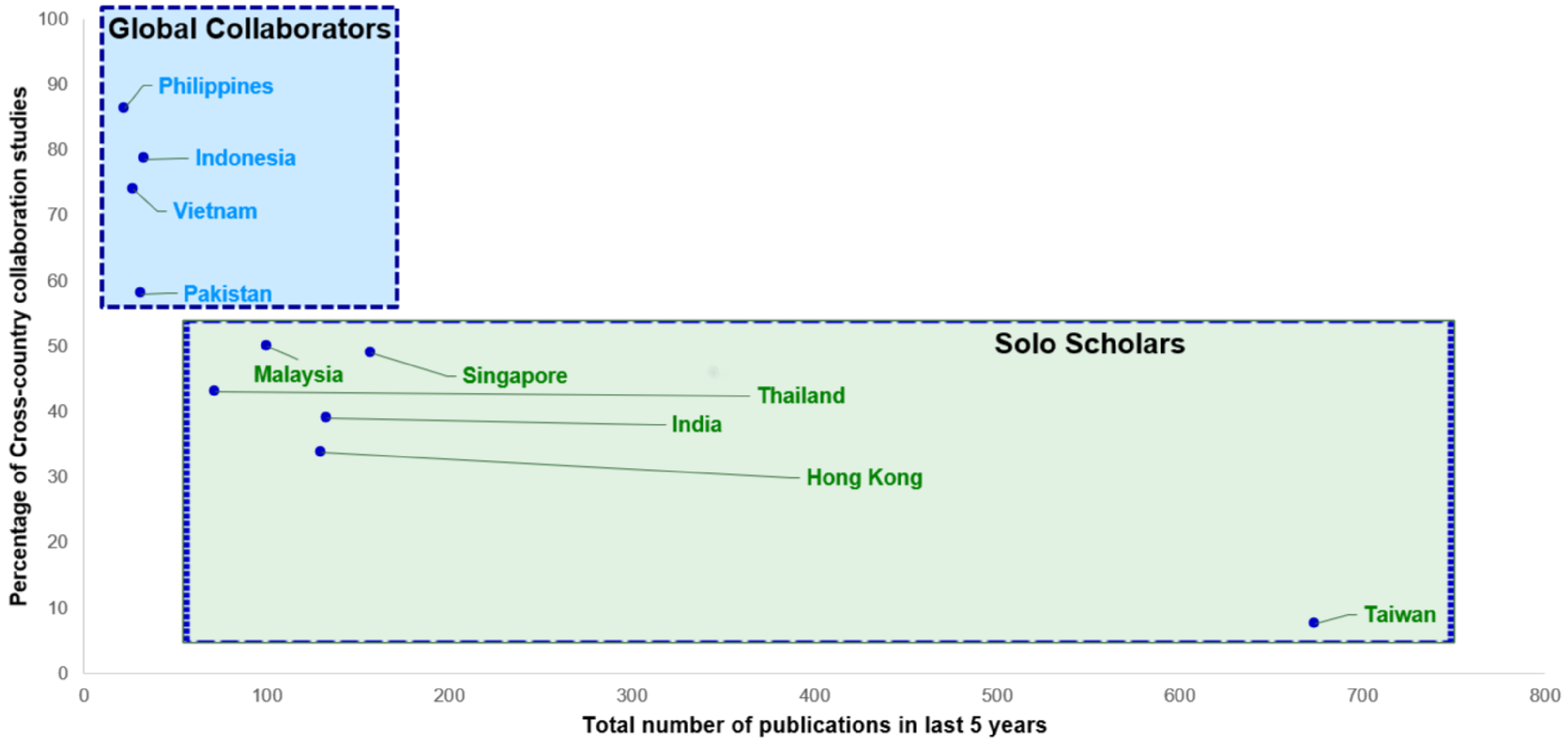

*Duplications in collaboration studies have been adjusted for the number of studies. Study period for Taiwan, India and Thailand: 2017 to 2022.[1] Study period for other countries: 2018 to 2023.[2]

1. Shau WY, Setia S, Chen YJ, Ho TY, Prakash Shinde S, Santoso H, et al. Integrated Real-World Study Databases in 3 Diverse Asian Health Care Systems in Taiwan, India, and Thailand: Scoping Review. J Med Internet Res. 2023 Sep 11;25:e49593. PMID: 37615085. doi: 10.2196/49593.
2. Shau WY, Santoso H, Jip V, Setia S. Integrated Real-World Data Warehouses Across 7 Evolving Asian Health Care Systems: Scoping Review. J Med Internet Res. 2024 Jun 11;26:e56686. PMID: 38749399. doi:10.2196/56686.
