# Supplementary material for: Bridging Real-World Data Gaps: Connecting Dots Across 10 Asian Countries
Source: JMIR Med Inform. 2024 Aug 15;12:e58548. doi: 10.2196/58548 (PMC11362708; doi:10.2196/58548)
Supplement: Multimedia Appendix 2 [file medinform_v12i1e58548_app2.pdf]

Multimedia Appendix 2

Bridging Real-World Data Gaps: Connecting Dots Across 10 Asian Countries

Guilherme Silva Julian, Wen-Yi Shau, Hsu-Wen Chou, Sajita Setia

Database types used in real-world studies across 10 target Asian countries

Clinical registries were the most common database source for CCCS in all countries [1, 2]. This preference for clinical registries can be attributed to several key factors, including, regulatory and ethical considerations that make them more feasible for standardization across borders compared to RWD databases [3] . Standardizing protocols across countries is more straightforward with registries as these databases are designed with these considerations from the outset, as opposed to EMR or claims databases, which may not primarily be structured for research use and can involve more varied regulatory landscapes [4, 5] .

Solo Scholars

Taiwan was shown to predominantly leverage health insurance/administrative claims databases for 73.5% (n=458/623) of its SCS publications [1], illustrating a robust sophisticated framework for medical reimbursement [6]. The comparatively lower use of EMRs/EHRs (11.4%) alongside a moderate reliance on clinical registries (36%) illustrates a prioritized approach to RWD collection, aiming for specific insights into healthcare utilization and costs [1]. It becomes evident that the country's approach is both pragmatic and effective, particularly in leveraging health insurance/administrative claims databases, highlighting the depth and maturity of Taiwan's health data ecosystem, which has been refined over more than two decades of claims data availability [7].

Singapore demonstrated a mixed usage of clinical registries and EMRs/EHR (57.5% and 53.8%, respectively) [2] while India showcased a balanced approach for SCS, significantly relying on EMRs/EHRs (n=45/81, 55.6%) and clinical registries

(n=38/81, 46.9%), reflecting its progress towards digital healthcare solutions [1]. Hong Kong adopted EMRs/EHRs as the principal data source (80.2%, n=69/86) for SCS [2]. This signifies a shift towards digital records for real-time, patient-centered data, facilitating a more dynamic approach to healthcare research and delivery [8]. Malaysia and Thailand demonstrated a strong preference for clinical registries in SCS (n=39/50, 78% and n=31/41, 75.6%, respectively) [1, 2], highlighting the availability and preference for detailed, condition-specific datasets that can provide deep insights into patient care pathways [9].

Global Collaborators

Indonesia and the Philippines also relied on clinical registries as the predominant warehouse for RWD generation over EMRs/EHRs (57.1% vs 28.6% and 66.7% vs 33.3%, respectively) [2]. Conversely, Pakistan and Vietnam displayed a higher utilization of EMRs/EHRs vs clinical registries (69.2% vs 23.1% and 57.1% vs 14.3%, respectively) [2], suggesting an evolving availability and accessibility of digital health records fit for healthcare research [10]. Surprisingly, the adoption of health insurance/administrative claims as the RWE data source was also more prominent for Vietnam among Global Collaborators (n=2/7, 28.6%) [2].

Abbreviations

CCCS: cross-country collaboration studies
EHR: electronic health record
EMR: electronic medical record
RWD: real-world data
RWE: real-world evidence
SCS: single-country studies
UHC: universal health coverage

References

1. Shau WY, Setia S, Chen YJ, Ho TY, Prakash Shinde S, Santoso H, et al. Integrated Real-World Study Databases in 3 Diverse Asian Health Care Systems in Taiwan, India, and Thailand: Scoping Review. J Med Internet Res. 2023 Sep 11;25:e49593. PMID: 37615085. doi: 10.2196/49593.
2. Shau WY, Santoso H, Jip V, Setia S. Integrated Real-World Data Warehouses across Seven Evolving Asian Healthcare Systems: A Scoping Review. J Med Internet Res. 2024 Jun 11;26:e56686. PMID: 38749399. doi: 10.2196/56686.
3. AHRQ Methods for Effective Health Care. In: Gliklich RE, Dreyer NA, Leavy MB, editors. Registries for Evaluating Patient Outcomes: A User's Guide. Rockville (MD): Agency for Healthcare Research and Quality (US); 2014.
4. Rumbold JM, Pierscionek B. The Effect of the General Data Protection Regulation on Medical Research. J Med Internet Res. 2017 Feb 24;19(2):e47. PMID: 28235748. doi: 10.2196/jmir.7108.
5. Hripcsak G, Albers DJ. Next-generation phenotyping of electronic health records. J Am Med Inform Assoc. 2013 Jan 1;20(1):117-21. PMID: 22955496. doi: 10.1136/amiajnl-2012-001145.
6. Lee P-C, Kao F-Y, Liang F-W, Lee Y-C, Li S-T, Lu T-H. Existing Data Sources in Clinical Epidemiology: The Taiwan National Health Insurance Laboratory Databases. Clinical Epidemiology. 2021 2021/03/01;13(null):175-81. doi: 10.2147/CLEP.S286572.
7. Sung SF, Hsieh CY, Hu YH. Two Decades of Research Using Taiwan's National Health Insurance Claims Data: Bibliometric and Text Mining Analysis on PubMed. J Med Internet Res. 2020 Jun 16;22(6):e18457. PMID: 32543443. doi: 10.2196/18457.

8. Tham TY, Tran TL, Prueksaritanond S, Isidro JS, Setia S, Welluppillai V. Integrated health care systems in Asia: an urgent necessity. Clin Interv Aging. 2018;13:2527-38. PMID: 30587945. doi: 10.2147/cia.S185048.

9. Lin LW AJ, Bayani DBS, Chan K, Choipel D, Isaranuwatchai W. Use of real-world data and real-world evidence to support drug reimbursement decision-making in Asia: A non-binding guidance document prepared by the REAL World Data in ASia for HEalth Technology Assessment in Reimbursement (REALISE) working group. Health Intervention and Policy Evaluation Research (HIPER), National University of Singapore. 2020. Available from: https://hiper.nus.edu.sg/wp-content/uploads/2021/03/REALISE-Full-guidance-post-feedback_20201211-version-1.1.pdf [accessed 2024-02-10].

10. Raman SR, O'Brien EC, Hammill BG, Nelson AJ, Fish LJ, Curtis LH, et al. Evaluating fitness-for-use of electronic health records in pragmatic clinical trials: reported practices and recommendations. J Am Med Inform Assoc. 2022 Apr 13;29(5):798-804. PMID: 35171985. doi: 10.1093/jamia/ocac004.
